# Decoding the gut interactome after severe trauma: from molecular dialogue to integrative treatment strategies

**DOI:** 10.3389/fmicb.2026.1885772

**Published:** 2026-07-29

**Authors:** Huiyu Huang, Min Yang, Lun Tan, Zhaojun Fu

**Affiliations:** 1 Trauma Center, Zigong Fourth People’s Hospital, Zigong, China; 2 Emergency Department, Zigong Fourth People’s Hospital, Zigong, China; 3 Neurology Department, Zigong Fourth People’s Hospital, Zigong, China; 4 Orthopaedic Center, Zigong Fourth People’s Hospital, Zigong, China

**Keywords:** gut microbiota, integrated treatment, interactome, pathobiome, pattern recognition receptors, severe trauma

## Abstract

Severe trauma constitutes not merely a localized injury, but a systemic insult to the host’s barrier systems driven by massive hemorrhage, hemorrhagic shock, and the systemic inflammatory response. Rather than being a passive victim of this cascade, the gut is widely considered a critical mediator contributing to the pathogenesis of remote MODS. The concept of the interactome redefines disease outcome as the product of a dynamic and reciprocal dialogue among host immune status, gut microbiota structure and function, and pathogen virulence expression. This framework offers a conceptual shift from the traditional single-pathogen model. Based on the interactome theory, this paper systematically reviews the current understanding of the evolution of intestinal microecology from symbiotic homeostasis to pathobiome after severe trauma. These include intestinal epithelial energy metabolism crisis induced by shock and hypoperfusion, excessive activation of pattern recognition receptors in the context of concurrent DAMPs and PAMPs, loss of mucosal protection associated with aryl hydrocarbon receptor ligand depletion, cytotoxic transformation suggested to be linked to bile acid metabolism disorder, and iatrogenic stress destruction of colonization resistance. On this basis, this paper outlines an integrated treatment strategy grounded in molecular targets. The strategy encompasses metabolic substrate supplementation with short-chain fatty acids and prebiotics, AhR signal reactivation via dietary tryptophan and phytochemicals, pathogenic group clearance and virulence inhibition using multi-targeted traditional Chinese medicine compounds, neuroimmune regulation through acupuncture, and a new clinical pathway combining dietary pre-rehabilitation with dynamic functional omics monitoring. Finally, we argue that future therapeutic strategies may benefit from shifting from pathogen eradication toward restoring host–microbiota symbiosis. This review provides a systematic framework for the precision intervention of post-traumatic gut microecology, spanning from molecular mechanisms to clinical translation.

## 1 Introduction

### 1.1 Systematic definition of major trauma

Traditional trauma medicine often focuses on the repair of local injuries such as craniocerebral injury, fracture and organ rupture. However, research in modern emergency and critical care medicine shows that severe trauma is an injury combined with massive blood loss and hemorrhagic shock, and its essence is a trigger event for a systemic disease (Pape et al., 2022). Trauma not only causes the destruction of anatomical structures, but also poses a systemic challenge to the barrier system of the whole body through three parallel pathological pathways: (1) systemic hypoperfusion and ischemia-reperfusion (I/R) injury caused by hemorrhagic shock; (2) Systemic inflammatory response syndrome (SIRS) is associated with endogenous danger signals released by tissue necrosis; (3) the second hit caused by bacterial translocation after intestinal barrier destruction (Lord et al., 2014). In this systemic hit, the gut is at the intersection of triple vulnerabilities due to its unique anatomical and physiological features (Eckburg et al., 2005). Its mucosal surface area is about 30–40 m^2^, which is the largest microbiota-host interface in human body. The villus microvessels were arranged in a hairpin pattern and were highly sensitive to hypoperfusion. It is home to about 10^13^ to 10^14^ microorganisms, and the total number of microbial genes is about 150 times that of the human genome (Eckburg et al., 2005).

### 1.2 The gut as the motor of remote organ dysfunction

Therefore, the gut after severe trauma is not only the affected organ, but also the motor driving the dysfunction of distant organs. Traumatic injury is frequently accompanied by a rapid disruption of the intestinal barrier, allowing large quantities of bacteria and endotoxins from the intestinal lumen to translocate into the portal vein and the systemic circulation. This process could propagate a local intestinal injury toward a systemic inflammatory storm that may ultimately contribute to distant organ damage. Previous studies have provided direct anatomical evidence that gut-derived bacteria can spread to brain tissue in patients who died of sepsis, which suggests a close association with neuroinflammation in septic encephalopathy (Singer et al., 2018). However, the use of anti-anaerobic antibiotics in ICU patients destroys intestinal colonization resistance, which is associated with dominant colonization by Enterobacteriaceae. This in turn is associated with an elevated risk of infectious complications and death, highlighting the protective value of an intact gut microbiota (Chanderraj et al., 2023). These two studies reveal a central conclusion that gut barrier integrity and microbiota homeostasis are critical lines of defense against distant organ dysfunction.

### 1.3 From dysbiosis to the gut interactome in trauma scenarios

Historically, the role of the intestinal microbiota in post-traumatic complications has been conceptualized through a reductive, descriptive lens. Pathological trajectories were often framed as linear sequences in which anatomical barrier failure was thought to allow mechanical bacterial translocation (Munley et al., 2023b; Ullah et al., 2024). This classical model, however, does not fully account for contemporary clinical paradoxes, such as localized or systemic infections arising in critically ill patients without detectable bacteremia despite rigorous antimicrobial prophylaxis (Merkow et al., 2015). To transcend these descriptive limitations, critical care medicine must integrate principles of systems biology. Although the term interactome is historically rooted in systems biology to describe global molecular interaction webs, its translation to the critical care arena fundamentally redefines surgical pathogenesis. Consequently, we propose to conceptualize the gut interactome under traumatic stress not as a static anatomical barrier, but as a specialized, tri-directional, and highly dynamic reciprocal network within which biochemical dialogues are continuously exchanged among host immunological sentinels, the functional metabolome of commensal microbes, and severe microenvironmental stressors (including hemorrhagic shock, ischemia-reperfusion injury, and volatile oxygen oscillations) (Cao et al., 2025).

The cornerstone of this trauma-specific interactome framework is that post-injury organ dysfunction and systemic infectious complications are not driven by any single entity in isolation, but emerge as systemic properties of dialogue breakdown among all three interconnected nodes (Cao et al., 2025). A key implication of this framework is that microbial virulence is re-conceptualized from its traditional status as an intrinsic, static genetic trait, and is re-conceptualized as an emergent phenotype dynamically elicited by host-microbe cross-talk (Casadevall and Pirofski, 2001). This phenotypic plasticity is supported by compelling empirical evidence. When exposed to host tissue stress or local nutrient depletion, opportunistic pathogens such as *Pseudomonas aeruginosa* intercept these endogenous chemical cues and could undergo a phenotypic switch suggestive of invasive, lethal phenotypes (Babrowski et al., 2013). Furthermore, indispensable intensive care interventions, such as anti-anaerobic antibiotics, opioid analgesics, and total parenteral nutrition, may inadvertently function as anthropogenic selective pressures that are associated with disruption of this delicate tri-directional equilibrium. This manifests as a therapeutic paradox that accelerates pathobiome maturation and interactome collapse (Cao et al., 2025).

### 1.4 Decoding molecular conversations

The molecular dialogue network between gut microbiota and host is the chemical basis for maintaining the homeostasis of interactome. Short-chain fatty acids (SCFAs) produced by obligate anaerobic fermentation of dietary fiber (Schulthess et al., 2019; Mukhopadhya and Louis, 2025), aryl hydrocarbon receptor (AhR) ligands produced by bacterial tryptophan metabolism (Qiu et al., 2012; Zelante et al., 2013), microbiota-mediated secondary bile acid conversion (Chen and Gong, 2025), and host Pattern Recognition Receptors (PRRs; Lavelle et al., 2010) continuously monitor microbial signaling together constitute an intricate multi-layered signaling network. Under the intense insult of severe trauma, shock-induced hypoperfusion and reperfusion injury simultaneously disrupt these molecular conversations at multiple key nodes. This drives a qualitative shift in the interactome from a health-promoting state to a pathogenic driver state (Munley et al., 2023b). This review adopts the interactome as its theoretical framework to systematically decode the mechanisms by which severe trauma disrupts the molecular dialogue between the intestinal microbiota and the host. Building on this mechanistic foundation, it proposes integrated, molecularly targeted treatment strategies. The overarching aim is to provide a systematic path from molecular mechanism to clinical translation for the precise intervention of intestinal microecology after trauma.

## 2 Physiological basis of gut microbiota-host molecular dialogue

### 2.1 SCFAs: metabolic mediators and epigenetic regulators

Under steady-state physiological homeostasis, SCFAs are not considered a homogenous metabolic pool. Instead, acetate, propionate, and butyrate display a notable degree of functional segregation with respect to their microbial origins and functional trajectories within the interactome. Luminal acetate and propionate are predominantly synthesized by members of the Bacteroidetes phylum through the wood-ljungdahl and succinate pathways, respectively, supplemented by specific *Bifidobacterium* species. In contrast, butyrate production is highly restricted to a specialized guild of obligate anaerobic Firmicutes. The most notable producers include *Faecalibacterium prausnitzii*, *Roseburia* spp., and *Eubacterium rectale*, which utilize the acetyl-CoA transferase/butyrate kinase pathway to ferment non-digestible carbohydrates (Schulthess et al., 2019; Wang et al., 2024).

Functionally, these three core metabolites contribute to local and systemic effects that are thought to support the baseline stability of the interactome network. Butyrate acts as the primary, non-redundant energetic substrate for the colonic epithelium, oxidized within colonocyte mitochondria to supply 60%–70% of their baseline cellular ATP requirements (Donohoe et al., 2011; Wang et al., 2024). Beyond its energetic role, butyrate acts as a powerful intracellular HDACs inhibitor, promoting the epigenetic differentiation of peripheral naive T cells into immunosuppressive Tregs to maintain mucosal immune tolerance (Qiu et al., 2012; Schulthess et al., 2019). Propionate largely circumvents local consumption, draining through the portal vein into the liver where it serves as a critical substrate for hepatic gluconeogenesis and interacts with hepatocyte GPR41/GPR43 receptors to downregulate hepatic lipogenesis. Acetate, boasting the highest luminal concentration and lowest hepatic clearance, readily enters the systemic circulation to reach peripheral tissues. There, it acts as a global metabolic and immunological rheostat, binding to GPR43 on circulating neutrophils and resident macrophages to modulate baseline leukocyte chemotaxis and suppress inappropriate pro-inflammatory cytokine shedding under healthy conditions (Abreu, 2010; Quintana and Sherr, 2013). Consequently, when severe traumatic stress later has been associated with the rapid eradication of these oxygen-sensitive obligate anaerobes, the asymmetric loss of these specific metabolite-driven signaling brakes may trigger a cascade of pathological events. An intracellular energy crisis rapidly ensues and may shift the interactome toward an uncontrolled inflammatory burst, as suggested by current experimental models (Peng et al., 2009; Donohoe et al., 2011).

### 2.2 Aryl hydrocarbon receptor: intracellular signaling hub of tryptophan metabolism in bacterial flora

The aryl hydrocarbon receptor (AhR) is a ligand-dependent transcription factor belonging to the basic helix-loop-helix/Per-Arnt-Sim (bHLH-PAS) superfamily. It is expressed in multiple mucosal immune populations, including intraepithelial lymphocytes, type 3 innate lymphoid cells (ILC3), Th17 cells, and dendritic cells (Quintana and Sherr, 2013). Gut microbiota, especially *Lactobacillus*, *Peptostreptococcus* and *Clostridium*, are known to produce a variety of AhR ligands through the tryptophan metabolic pathway. It mainly includes indole-3-acetic acid (IAA), indole-3-propionic acid (IPA), indole-3-aldehyde (IAld) and indole-3-acetaldehyde (IAAld; Zelante et al., 2013). In the ligand-free state, AhR forms cytoplasmic complexes with molecular chaperones such as heat shock protein 90 (HSP90), XAP2 and p23. Following ligand binding, AhR is reported to undergo a conformational change and subsequently translocate to the nucleus. There, it forms a heterodimer with the aryl hydrocarbon receptor nuclear translocator (ARNT). The AhR–ARNT complex then binds xenobiotic response elements (XREs) in the promoter regions of target genes, initiating the transcriptional program (Murray et al., 2014). In ILC3s, AhR signaling drives the transcription and secretion of IL-22. This cytokine has been shown to stimulate Paneth cells to secrete C-type lectin antimicrobial peptides such as Reg3γ and Reg3β. It also induces intestinal epithelial cells to produce mucus-associated proteins and enhances the integrity of tight junctions (Qiu et al., 2012). Studies in germ-free mice and AhR gene-null mice have reported severe intestinal epithelial barrier dysfunction, insufficient number and function of ILC3, decreased IL-22 expression, decreased secretion of antimicrobial peptides, and significantly increased susceptibility to intestinal pathogens, such as *Citrobacter rodentium* (Kiss et al., 2011). In addition, gut microbiota tryptophan metabolism is also suggested to interact with the host 5-hydroxytryptamine (5-HT) system. Enterochromaffin cells are the main synthesis site of 5-HT in the human body. In particular, SCFAs have been shown to induce the expression of tryptophan hydroxylase 1 (Tph1) in enterochromaffin cells through GPR43 signaling, regulate the synthesis rate of 5-HT, and then affect the intestinal peristaltic reflex, secretory function and visceral sensation (Yano et al., 2015).

### 2.3 PRRs: immune sensors and spatial polarity layout

The maintenance of immune tolerance alongside rapid pathogen vigilance within the gut interactome is fundamentally governed by the strict spatial polarity and functional division of PRRs across the intestinal epithelial barrier. Transmembrane Toll-like receptors (TLRs) and cytoplasmic Nod-like receptors (NLRs) form a tightly compartmentalized surveillance network segregated between the apical and basolateral cellular domains (Lord et al., 2014; Ullah et al., 2024). Under steady-state physiological conditions, expression of pro-inflammatory TLRs, most notably TLR4, is virtually absent or transcriptionally silenced at the apical brush border facing the lumen (Abreu, 2010). This apical silencing constitutes a primary evolutionary adaptation that prevents continuous, detrimental inflammatory responses to the massive background load of luminal commensal pathogen-associated molecular patterns (PAMPs). Conversely, functional TLR4, along with the flagellin sensor TLR5, is strategically sequestered at the basolateral membrane domain, while NLRs (such as NOD1 and NOD2) are localized exclusively within the cytoplasmic matrix, creating a multi-tiered secondary defense perimeter (Poltorak et al., 1998; Ullah et al., 2024).

This spatial partitioning is believed to underpin a sophisticated, dual-input activation architecture that integrates both exogenous PAMPs and endogenous damage-associated molecular patterns (DAMPs). Under healthy homeostasis, basolateral TLRs and cytoplasmic NLRs sample only trace amounts of translocated bacterial components, are thought to transmit low-level, tonic signals that support physiological immuno-training and reinforce tight junction integrity without igniting inflammatory cascades (Ullah et al., 2024; Walrath et al., 2024). However, this spatial equilibrium is considered to render the interactome highly vulnerable to cooperative PAMP/DAMP hyperactivation upon barrier breach. When physical tissue injury or ischemic necrosis leads to the release of host DAMPs (such as mitochondrial DNA, extracellular ATP, and HMGB1) into the lamina propria, these DAMPs are suggested to prime resident macrophages and dendritic cells (Lord et al., 2014; Walrath et al., 2024). Simultaneously, the concurrent disintegration of the paracellular barrier is associated with a massive influx of luminal PAMPs (primarily LPS) to flood the basolateral compartment (Jiang et al., 1995). This combined surge encounters the highly exposed basolateral TLR4 and intracellular NLR networks. The simultaneous docking of interleaved PAMPs and DAMPs induces robust, cooperative recruitment of both MyD88 and TRIF adaptor proteins, and is thought to generate a synergistic signaling amplification that promotes massive NF-κB nuclear translocation and may precipitate an uncontrolled local and systemic cytokine storm (Poltorak et al., 1998; Walrath et al., 2024).

### 2.4 Bile acids: multiorgan signaling axis mediated by microbiota

Primary bile acids are synthesized from cholesterol catalyzed by CYP7A1 and CYP27A1 in the liver and excreted into the duodenum through the bile duct (Fuchs et al., 2025). In the distal ileum and colon, the gut microbiota, particularly the genera *Clostridium*, *Bacteroides*, *Lactobacillus*, and *Bifidobacterium*, deconjugate taurine or glycine through Bile salt hydrolase (BSH) activity to generate free primary bile acids. These free primary bile acids are then converted to secondary bile acids, catalyzed by 7α-dehydrogenase (Ridlon et al., 2006).

Gut-liver axis, FXR-FGF19 negative feedback loop. As a high-affinity ligand of farnesoid X receptor (FXR), secondary bile acids are reported to possess stronger signaling activity compared with primary bile acids (Chiang and Ferrell, 2022). Activation of intestinal FXR induces ileal epithelial cells to secrete fibroblast growth factor 19 (FGF19). FGF19 travels via the portal vein to the liver, where it inhibits CYP7A1 transcription. This intestine–liver negative feedback loop maintains bile acid pool homeostasis (Katafuchi and Makishima, 2022). This transformation is considered an important step for cholesterol clearance and lipid digestion and absorption. It is frequently cited as a classic model of bacterial reprogramming of host metabolic signals.

Gut-immune axis, TGR5-mediated anti-inflammatory effect. Secondary bile acids are also potent agonists of G protein-coupled receptor 5 (TGR5). TGR5 is widely expressed in intestinal macrophages, dendritic cells and enteroendocrine L cells (Yan et al., 2025). In macrophages, the activation of TGR5 inhibits the assembly and activation of NLRP3 inflammasome through the cAMP-PKA pathway, reduces the mature secretion of IL-1β and IL-18, and is thought to exert a significant anti-inflammatory effect. At the same time, TGR5 signaling promotes the secretion of glucagon-like peptide-1 (GLP-1) by intestinal endocrine L cells, which not only regulates insulin secretion and satiation, but also exerts a paracrine anti-inflammatory effect on intestinal immune cells. In the context of uncontrolled intestinal inflammation after trauma, diminished TGR5 signaling may contribute to macrophage inflammatory polarization. Notably, bile acids themselves are also suggested to serve as regulators of gut microbiota composition. Their inherent detergent activity can directly damage the cell membrane integrity of susceptible strains, thereby exerting screening pressure on the bacterial population. Thus, microbiota BSH and 7α-dehydrogenase activity essentially may constitute a self-shaping feedback loop. Microbiota metabolize bile acids to change the composition and antibacterial activity of the intestinal bile acid pool, and then reverse screen out strains that can tolerate a specific bile acid spectrum (Islam et al., 2011).

### 2.5 Multi-level microbiota regulation of intestinal barrier function

The synergistic effects of the above molecular dialogue axes are thought to converge toward the precise regulation of intestinal barrier function. Mucus layer, a polymer network formed by serine protease decomposition and unfolding of MUC2 secreted by goblet cells, constitutes the first molecular interface between host and microbiota (Johansson et al., 2013). Germ-free mice have been reported to exhibit a markedly thinned colonic mucus layer coupled with a reduced degree of MUC2 polymerization. Conversely, colonization with butyric acid-producing bacteria (such as *Faecalibacterium prausnitzii*) or direct butyrate supplementation has been shown to restore mucosal thickness and function by elevating H3K9 acetylation levels within the MUC2 gene promoter region (Johansson et al., 2015). Under the mucus layer, the tight junction complex formed by claudin family, occludin, ZO-1, JAM-A and other proteins between intestinal epithelial cells is subject to continuous dynamic regulation by microbial signals. Butyric acid has been shown to promote the transport of ZO-1 and occludin to the cell membrane through AMPK pathway phosphorylation and enhances tight junction assembly (Peng et al., 2009). Indole AhR ligands are suggested to optimize paracellular permeability by up-regulating the expression of multiple claudin isoforms (Yu et al., 2018). In the lamina propria, microbiota-trained Treg cells and Th17 cells maintain the delicate balance between immune tolerance and defense. Treg cells limit the excessive inflammatory response to commensal bacteria by secreting IL-10, while Th17 cells and their secreted IL-17 and IL-22 enhance the antimicrobial defense of the epithelium. Any deviation of this balance, either a dysregulation of Treg to Th17 switching or a change in the IL-22/IL-10 ratio, has been associated with uncontrolled intestinal inflammation and collapse of interactome homeostasis (Honda and Littman, 2016).

## 3 Pathological reprogramming of the gut interactome following severe trauma

The clinical trajectory of severe trauma is deeply intertwined with the hyper-acute destabilization of the intestinal microenvironment. Under the intense insult of systemic hypoperfusion and subsequent resuscitation, the delicate molecular dialogues that sustain healthy interactome homeostasis are simultaneously disrupted across multiple synchronized nodes. This section systematically decodes the pathological transition of the post-traumatic gut, tracing the macro-environmental forces that are thought to drive pathobiome evolution, deconstructing the targeted collapse of the four core molecular signaling axes, mapping the dual-channel propagation of distant organ injury, and contextualizing the iatrogenic pressures that act as a detrimental second hit.

### 3.1 Macro-environmental shifts and pathobiome evolution

Severe trauma represents not only a disruption of anatomical structures but also a profound ecological disturbance that fundamentally reconfigures the intestinal microenvironment (Lord et al., 2014; Pape et al., 2022). Under homeostatic conditions, the intestinal epithelium sustains a deeply anaerobic environment through controlled oxygen consumption, thereby supporting a commensal community dominated by obligate anaerobes of the Bacteroidetes and Firmicutes phyla (Zheng et al., 2015; Wang et al., 2024). However, trauma-induced shock and hypoperfusion disrupt this equilibrium (Eckburg et al., 2005; Klingensmith and Coopersmith, 2016).

The violent oscillation in luminal oxygen partial pressure (pO_2_) that accompanies resuscitation is proposed to function as a potent ecological filter, as suggested by experimental models of resuscitation (Libert et al., 2017). During hemorrhagic shock, the oxygen supply to intestinal epithelial cells is profoundly interrupted (Zheng et al., 2015). Subsequently, clinical interventions, namely fluid resuscitation and vasoactive drug administration, induce intermittent reperfusion, is associated with violent swings in luminal pO_2_ from below 5 mmHg to above 50 mmHg (Libert et al., 2017). This redox instability has been interpreted as an ecological filter. Obligate anaerobes, which lack robust antioxidant defenses, are reported to be depleted, thereby potentially creating a permissive niche for the rapid expansion of facultative anaerobes that are equipped with robust oxidative-stress responses, including *Pseudomonas aeruginosa*, *Enterococcus* spp., and Enterobacteriaceae (Litvak et al., 2017; Chen et al., 2022; Sun et al., 2024; Zünd et al., 2025). The resulting community has been defined as the pathobiome, defined as a disease-associated microbial assemblage that emerges not through the introduction of exogenous pathogens but through the environmental screening of the endogenous commensal pool (Mittal and Coopersmith, 2014; Cao et al., 2025).

Concurrently, this ecological transition is thought to be accelerated by host-to-microbe chemical communication (Babrowski et al., 2012, 2013). Endogenous stress signals released into the gut lumen by the traumatized host, including catecholamines and endogenous opioids, could be sensed by sensory machinery on the surface of opportunistic pathogens. This host-to-microbe signaling is further amplified by clinically administered opioids such as morphine (Babrowski et al., 2012, 2013). This host-to-microbe information transfer has been reported to induce a phenotypic virulence switch. *Pseudomonas aeruginosa*, for instance, senses these host-derived chemical cues and transitions from a commensal lifestyle to a hypervirulent mode, characterized by the upregulation of elastase (LasB), pyocyanin production, and robust biofilm formation (Williams et al., 2000; Babrowski et al., 2012).

### 3.2 Targeted deconstruction of the four core molecular dialogue axes

**FIGURE 1:**
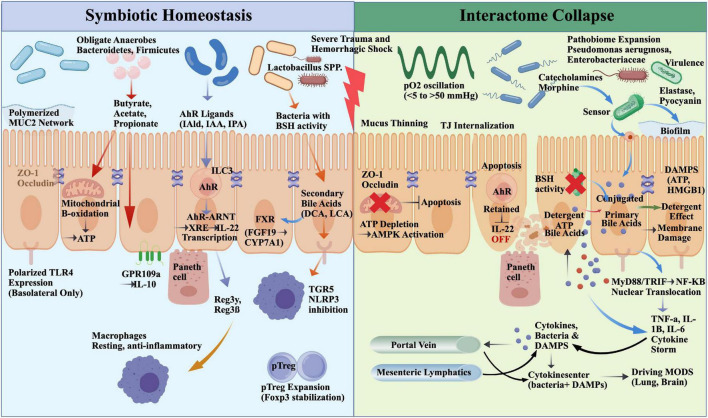
Homeostatic vs. pathological gut interactome dialogue after severe trauma. A comparative cross-section illustration of the intestinal epithelial barrier in symbiotic homeostasis (left panel) versus post-traumatic interactome collapse (right panel), separated by a traumatic trigger event. Symbiotic Homeostasis: The deeply anaerobic lumen supports dominant obligate anaerobes (Bacteroidetes, Firmicutes). An intact mucus layer covers healthy columnar epithelial cells with continuous tight junctions (ZO-1, Occludin). Distinct molecular dialogues maintain health: SCFA Axis, AhR Axis, Bile Acid Axis and PRR Tolerance. Interactome Collapse: The severe trauma microenvironment is associated with a profound ecological and metabolic disruption, which has been conceptualized as a systemic engine of MODS: Ecological Shift, Barrier Disassembly, Halted Dialogues and Pathological Hyperactivation. The mechanistic link between the two states is established through four interconnected molecular axes-SCFA, AhR, bile acid, and PRR-whose synergistic interactions determine the trajectory of the gut interactome. Under homeostasis (left), SCFAs and AhR ligands cooperate to maintain epithelial barrier integrity and immune tolerance, while secondary bile acids suppress NLRP3 inflammasome activation via TGR5. These pathways converge to reinforce a mutualistic dialogue that prevents PRR hyperactivation. Upon severe trauma (right), synchronized collapse across all four axes, driven by ischemia, microbial depletion, and metabolic failure, amplifies PAMP/DAMP signaling and tips the balance toward pathological inflammation. This multi-axial crosstalk, rather than any single pathway in isolation, defines the functional state of the gut interactome. In this framework, the gut interactome encompasses the full tri-directional signaling network among host immunity, microbiota metabolism, and microenvironmental stress; pathobiome denotes the disease-associated microbial community selected from endogenous commensals under environmental pressure; and dysbiosis is a descriptive term for compositional alterations that may or may not progress to pathobiome configuration. Created with BioGDP.com (Jiang et al., 2025).

The collapse of gut function may be viewed, in essence, as a destabilization and reprogramming of the bidirectional dialogue among host immune status, the functional architecture of the microbiota, and pathogen virulence expression (Deitch, 2010; Cao et al., 2025), as schematically contrasted in Figure 1, which highlights the synergistic interactions among the four molecular axes, SCFA, AhR, bile acid, and PRR, rather than depicting them as isolated pathways.

#### 3.2.1 SCFA depletion and the epithelial energy crisis

Under shock-induced hypoperfusion, mitochondrial oxidative phosphorylation is substantially impaired in preclinical models; consequently, colonocytes are thought to experience severe energy starvation (Donohoe et al., 2011). This systemic hypoxic state is associated with suppression of the metabolic β-oxidation of butyrate, the chief energy substrate normally supplying 60%–70% of enterocyte requirements (Donohoe et al., 2011; Wang et al., 2024). The acute depletion of luminal SCFAs, which is attributed to the loss of obligate anaerobic fermenters, is considered to deprive the host epithelium of its primary metabolic fuel (Schulthess et al., 2019; Mukhopadhya and Louis, 2025). The ensuing depletion of ATP is thought to trigger aberrant activation of the AMPK pathway, leading to pathological phosphorylation and internalization of tight junction proteins (Peng et al., 2009). The resultant dislocation of occludin and ZO-1 from the cell membrane to the cytoplasm has been associated with a sharp increase in paracellular permeability (Deng et al., 2026). In models of hemorrhagic shock, this disruption of the mucosal barrier has been observed to be accompanied by a significant increase in intestinal epithelial cell apoptosis and a complete destabilization of the mucus layer (Rupani et al., 2007). Clinically, markers of enterocyte injury such as intestinal fatty acid-binding protein (I-FABP) become significantly elevated in patients with severe trauma within a short time after injury, characteristically demonstrating the rapid onset of critical illness-induced gut disruption (de Haan et al., 2009).

#### 3.2.2 AhR ligand starvation and mucosal immune silencing

The drastic perturbation of intestinal flora by severe trauma leads to a substantial reduction in tryptophan-metabolizing flora, with the depletion of *Lactobacillus* and *Clostridium* being particularly prominent (Munley et al., 2023b; Sun et al., 2023). The abundance of these genera has been reported to decrease significantly within 24 h after trauma. This is accompanied by a profound depletion of luminal AhR ligands, including IAld and IAA, and is correlated with a marked decline in AhR transcriptional activity (Sun et al., 2023). Under physiological conditions, binding of these ligands induces AhR to translocate to the nucleus, where it forms a heterodimer with ARNT to bind the XRE in the IL-22 gene promoter, driving IL-22 transcription in type ILC3s (Qiu et al., 2012; Murray et al., 2014). In the setting of AhR ligand depletion, AhR is suggested to be retained within the cytoplasm, which correlates with a substantial reduction in IL-22 transcription and secretion (Sun et al., 2023). In the absence of IL-22 signaling, Paneth cells are reported to exhibit reduced capacity to secrete C-type lectin antimicrobial peptides such as Reg3γ and Reg3β, while MUC2 mucin synthesis decreases, which may cause the protective mucus layer to thin, fragment, and lose its fundamental defense barrier (Sonnenberg et al., 2011).

#### 3.2.3 Pathological PRR hyperactivation and combined PAMP/DAMP burst

The breakdown of the physical interactome barrier is proposed to shift PRR signaling from physiological immune training toward a destructive pathological burst (Ullah et al., 2024). Alexander and colleagues reported that extensive cell destruction caused by severe trauma is associated with the release of a large number of DAMPs into the lamina propria. These include extracellular ATP, mitochondrial DNA (mtDNA), and high mobility group box 1 (HMGB1), which activate innate immunity via TLR and NLR pathways (Lord et al., 2014; Walrath et al., 2024). Simultaneously, barrier breakdown has been shown to allow translocating pathogen-associated molecular patterns (PAMPs), primarily LPS, to gain access across the disrupted paracellular space (Jiang et al., 1995).

This combined surge is believed to engage TLR4. Under steady-state conditions, TLR4 expression at the apical brush border is extremely low to ensure immune tolerance (Abreu, 2010). However, when the barrier is breached, LPS gains access to the high density of TLR4 pathologically exposed on the basolateral surface of enterocytes and resident macrophages (Poltorak et al., 1998). This interaction triggers robust activation of both MyD88-dependent and TRIF-dependent signaling cascades, which are thought to promote massive NF-κB nuclear translocation and contribute to an uncontrolled local and systemic cytokine storm consisting of TNF-α, IL-1β, and IL-6 (Poltorak et al., 1998). This transition of PRR signaling is considered a key factor in the evolution of post-traumatic SIRS toward sepsis (Ullah et al., 2024; Walrath et al., 2024).

#### 3.2.4 Bile acid dysmetabolism and cytotoxic transformation

Traumatic stress is associated with disruption of the microbiota-bile acid metabolic dialogue, potentially via multiple synchronized mechanisms. Splanchnic hypoperfusion may lower the luminal pH below 6.0 due to severe tissue acidosis, a condition that has been shown to compromise the catalytic efficiency of BSH, whose optimal functional window is between pH 5.5 and 6.5 (Ridlon et al., 2006). This enzymatic inhibition is thought to be further exacerbated by the antibiotic-driven eradication of 7α-dehydrogenase-encoding genera (such as *Clostridium* clusters), which is associated with an almost complete interruption of secondary bile acid synthesis (Ridlon et al., 2006; Kullberg et al., 2025). Decreased BSH activity is correlated with a substantial accumulation of conjugated primary bile acids in the colon. Due to their inherent hydrophobicity, these primary bile acids are reported to exert a detergent effect on the host tissue. They embed directly into the lipid bilayer of epithelial membranes, dissolving cell structures, which is thought to amplify enterocyte apoptosis, and potentially perpetuating the gut interactome in a cytotoxic downward spiral (Islam et al., 2011).

### 3.3 Dual-channel conduction of remote organ injury: the gut-lymph axis

**FIGURE 2:**
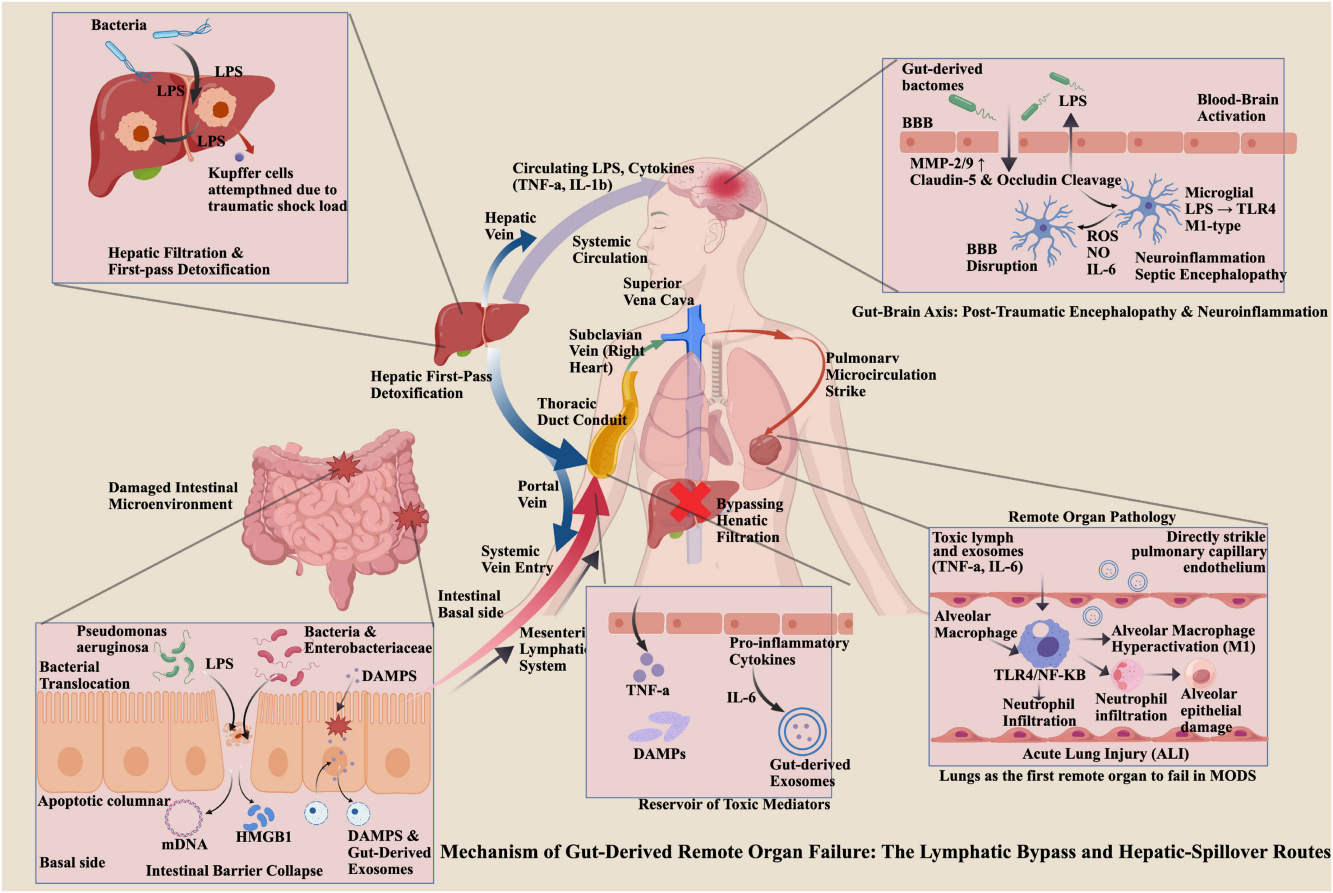
Dual-channel pathways of remote organ injury: the gut as the MODS engine. A flat anatomical flowchart mapping the systemic translocation and target-organ polarization of gut-derived pathogenic factors following severe trauma, highlighting the mesenteric lymph path as a hypothesized non-hepatic anatomical bypass. Importantly, these two pathways carry substantially different levels of evidence: the portal vein route is well-established in human studies, whereas the gut-lymph axis is a hypothesis derived primarily from rodent models (see section 3.3 Dual-channel conduction of remote organ injury: the gut-lymph axis for detailed discussion). Source: Damaged Gut: Severe trauma breaks the intestinal barrier, triggering epithelial apoptosis and widened intercellular junctions. This breach permits the mass translocation of pathogens (*Pseudomonas aeruginosa*, Enterobacteriaceae) and LPS, alongside the basal release of tissue DAMPs (mtDNA, HMGB1) and pathological gut-derived exosomes. Pathway A: Portal Vein Route (Traditional Hepatic Axis-Well-Established in Humans): Translocated bacteria and LPS enter the portal vein to undergo hepatic first-pass filtration. Under traumatic shock loads, Kupffer cells may become partially overwhelmed, allowing residual endotoxins to enter the systemic circulation, disrupt the blood-brain barrier (BBB), and activate microglia to drive neuroinflammation and septic encephalopathy. This pathway is supported by direct human evidence, including detection of gut-derived bacterial components in systemic circulation and correlation with inflammatory markers. Pathway B: Gut-Lymph Axis Route (Hypothesized Non-Hepatic Bypass-Based on Animal Models): Pro-inflammatory cytokines (TNF-α, IL-6), DAMPs, and exosome cargoes are hypothesized to circumvent the liver by entering the mesenteric lymphatics. In rodent models, this toxic reservoir travels through the thoracic duct and enters the systemic circulation via the subclavian vein, hypothetically delivering pathological mediators directly to the pulmonary microcirculation. Remote Organ Failure: Upon reaching the lungs, lymph-transported DAMPs and exosomes are reported to activate alveolar macrophages toward pro-inflammatory M1 polarization via TLR4/NF-κB signaling in animal models. This molecular cascade is associated with massive neutrophil infiltration and alveolar epithelial damage, which may contribute to Acute Lung Injury (ALI) and has been proposed to establish the lungs as a primary remote organ to fail during gut-driven MODS. However, direct human evidence for the gut-lymph axis is currently lacking, and the relative contribution of this pathway versus the portal route in human trauma remains an open question (see section “6.4 Controversies and unresolved issues in trauma research”). Created with BioGDP.com (Jiang et al., 2025).

The systemic propagation of gut-derived pathogenic factors following severe trauma is hypothesized to occur through two distinct anatomical conduits: the portal venous route and the mesenteric lymphatic system. The anatomical layout of these two conduits and their respective target organs is schematically illustrated in Figure 2.

Pathway A: Portal Vein Route (Well-Established in Humans). Under the traditional hepatic route, translocated bacteria and high concentrations of LPS enter the portal vein to undergo hepatic first-pass filtration (Jiang et al., 1995). Under traumatic shock loads, Kupffer cells may become partially overwhelmed, allowing residual endotoxins to enter the systemic circulation, disrupt the blood–brain barrier (BBB), and activate microglia to drive neuroinflammation and septic encephalopathy (Jiang et al., 1995; Singer et al., 2018). This pathway is supported by direct evidence from human studies, including the detection of gut-derived bacterial DNA in brain tissue of sepsis patients and the correlation between portal endotoxin levels and systemic inflammatory markers (Jiang et al., 1995; Singer et al., 2018).

Pathway B: Gut-Lymph Axis Route (Hypothesis Based Primarily on Animal Models). Beyond the portal venous route, the mesenteric lymphatic system has been proposed as a non-hepatic conduit that may accelerate the progression from local intestinal insult to systemic organ failure (Deitch, 2010; Ma et al., 2021). This hypothesis originates from rodent models of hemorrhagic shock and intestinal ischemia–reperfusion, in which pro-inflammatory cytokines, necrotic DAMPs, and gut-derived exosomes carrying pathological cargo signatures have been shown to preferentially enter the mesenteric lymphatics rather than the portal vein, and travel via the thoracic duct to the systemic circulation, directly reaching the pulmonary microcirculation (Kojima et al., 2018; Ma et al., 2021). Because mesenteric lymph flows through the thoracic duct and enters the systemic circulation directly via the subclavian vein, it completely circumvents hepatic filtration and first-pass detoxification (Deitch, 2010; Ma et al., 2021). This toxic reservoir has been associated with acute endothelial activation and the polarization of resident alveolar macrophages toward a pro-inflammatory M1 phenotype via TLR4/NF-κB signaling, promoting subsequent neutrophil infiltration (Deitch, 2010).

Clinical uncertainty and translational gap. It is important to emphasize that the gut-lymph axis has been characterized almost exclusively in rodent models of hemorrhagic shock and intestinal ischemia-reperfusion. Direct evidence for this pathway in human trauma patients is currently lacking, as sampling mesenteric lymph or isolating intact, gut-derived exosomes from circulating blood under acute resuscitation conditions presents substantial ethical and logistical challenges. The relative contribution of the lymphatic route versus the portal route to systemic inflammation and remote organ injury in human trauma remains an open question. Therefore, while the gut-lymph axis offers a compelling mechanistic hypothesis to explain the pulmonary injury patterns observed in gut-driven MODS, particularly the observation that the lungs are often the first remote organ to fail, its clinical significance should be interpreted with appropriate caution until validated in human studies. Nevertheless, the hypothesis has generated testable predictions that may inform future translational research, including the identification of lymphatic-specific biomarkers and the evaluation of thoracic duct drainage as a potential therapeutic strategy in animal models (Deitch, 2010).

### 3.4 The gut-brain axis: systemic spillover and post-traumatic neuroinflammation

Beyond localized hepatic and pulmonary complications, the systemic propagation of un-degraded, gut-derived toxic mediators establishes a critical pathological bridge to the central nervous system via the gut-brain axis (Jiang et al., 1995; Singer et al., 2018). When circulating LPS, pro-inflammatory cytokines (TNF-α, IL-1β), and cytotoxic primary bile acids may overwhelm the shock-paralyzed hepatic Kupffer cell filtration barrier, they are thought to gain access to the systemic circulation and reach the cerebral vasculature (Jiang et al., 1995). This systemic challenge has been associated with the upregulation and secretion of matrix metalloproteinases (specifically MMP-2 and MMP-9) in brain microvascular endothelial cells. These enzymes are thought to mediate the enzymatic cleavage of claudin-5 and occludin, potentially compromising the physical integrity and sealing capacity of BBB (Jiang et al., 1995). This hyper-permeable state is believed to allow circulating endotoxins to gain access to the immunologically privileged cerebral parenchyma and directly bind TLR4 complexes on resting microglia (Jiang et al., 1995; Singer et al., 2018). This interaction could activate the intracellular MyD88/NF-κB signaling cascade, which is thought to drive rapid microglial polarization into a hyperactive M1 pro-inflammatory phenotype. The subsequent localized burst of neurotoxic mediators, including reactive oxygen species (ROS), nitric oxide (NO), and IL-6, profoundly impairs synaptic plasticity and triggers neuronal apoptosis, potentially contributing to the pathogenesis of post-traumatic neuroinflammation and septic encephalopathy (Jiang et al., 1995; Singer et al., 2018), with the anatomical pathways and multi-organ impacts comprehensively mapped in Figure 2.

### 3.5 Iatrogenic stress: the second hit under the therapeutic paradox

In clinical practice, many indispensable therapeutic interventions are themselves regarded as intense selective pressures on the gut interactome, which may inadvertently drive the microbiota toward pathological phenotypic evolution (Dethlefsen et al., 2008; Cao et al., 2025).

#### 3.5.1 Comparative impact of therapeutic interventions on the interactome

To more clearly elucidate how iatrogenic factors reshape the intestinal microenvironment, Table 1 summarizes the molecular effects of three core interventional modalities.

**TABLE 1:** Dynamic impact of iatrogenic interventions on the gut interactome.

Iatrogenic intervention	Direct effects on the microbiota	Dynamic consequences for the interactome	References
Broad-spectrum antibiotics	Rapidly reduces α-diversity and indiscriminately eliminates anaerobic commensals	Eradicates colonization resistance, opening an ecological niche for the opportunistic expansion of Enterobacteriaceae; increases the risk of sepsis within 90 days of discharge	Dethlefsen et al., 2008; Chanderraj et al., 2023; Kullberg et al., 2025
Opioid analgesics (morphine)	Alters intestinal motility and secretory environment	Acts as an exogenous inductive signal that activates the virulence regulatory network of pathogens such as *Pseudomonas aeruginosa*, upregulating elastase LasB and biofilm formation, and precipitating gut-derived sepsis	Babrowski et al., 2012
Total parenteral nutrition (TPN)	Deprives the microbiota of metabolic substrates, specifically dietary fiber and polysaccharides	Induces ecological starvation, forcing Proteobacteria to utilize host mucin as a carbon source and accelerating mucus layer depletion; reduces microbial diversity, resulting in the overgrowth of Proteobacteria	Miyasaka et al., 2013; Neag et al., 2025

#### 3.5.2 Virulence reprogramming triggered by iatrogenic signals

Iatrogenic pressures reprogram the commensal microbiota toward an invasive phenotype by fundamentally altering the nature of the dialogue within the interactome. The ecological cleansing effect of antibiotics is exemplified by the fact that the use of anti-anaerobic antibiotics, such as clindamycin and metronidazole, is associated with the irreversible loss of obligate anaerobic commensals. This represents not only a reduction in bacterial load, but also the complete dismantling of the chemical defense system within the gut that senses and suppresses pathogen virulence (Chanderraj et al., 2023; Kullberg et al., 2025). Morphine, in turn, has been reported to act as a stimulant for pathogens in experimental models. Beyond exerting its analgesic effects through the central nervous system, it can cross the biological boundary to function as a signaling molecule sensed by pathogenic bacteria (Babrowski et al., 2012). In the context of an intestinal barrier already compromised by trauma, the administration of morphine may inadvertently function as a contributing factor that shifts the interactome toward sepsis. The mucus-eroding effect of total parenteral nutrition is equally noteworthy. By completely bypassing the gut, TPN is thought to expose the microbial community to an energy crisis. To survive, bacteria capable of degrading host mucins, such as certain Proteobacteria, gain a competitive advantage. This mucin-degrading activity of the host’s physical barrier may further accelerate the collapse of the interactome (Miyasaka et al., 2013; Neag et al., 2025).

#### 3.5.3 Balancing perspective: necessity versus risk

The preceding discussion has highlighted the microbiome-disrupting effects of broad-spectrum antibiotics, opioid analgesics, and total parenteral nutrition. However, it is equally important to acknowledge that these interventions remain indispensable in the acute management of severely traumatized patients. Antibiotics are essential for the prevention and treatment of life-threatening infections in the context of open wounds, visceral injuries, and invasive procedures (Lord et al., 2014; Pape et al., 2022); opioid analgesics are often necessary for adequate pain control, particularly in patients with hemodynamic instability where alternative analgesic modalities may be contraindicated (Babrowski et al., 2012); and TPN provides crucial nutritional support when enteral access is surgically or functionally precluded (Miyasaka et al., 2013). The therapeutic paradox described here is not an argument against their use, but rather a call for judicious application and active mitigation of their unintended consequences.

A balanced risk-benefit assessment should consider both the acute life-saving benefits and the delayed ecological consequences of these interventions. Potential mitigation strategies include antibiotic stewardship programs to minimize unnecessarily prolonged or anti-anaerobic coverage (Kullberg et al., 2025), multimodal analgesic protocols to reduce opioid exposure while maintaining adequate pain control (Babrowski et al., 2012), and early enteral nutrition whenever feasible to maintain microbial metabolic function and barrier integrity (Miyasaka et al., 2013). The clinical challenge lies in individualizing these approaches that recognizing that the optimal balance between immediate therapeutic necessity and long-term ecological preservation may vary substantially across patients based on injury severity, hemodynamic status, and baseline microbiome resilience. This nuanced perspective, rather than a binary view of iatrogenic interventions as either wholly beneficial or detrimental, is essential for the practical application of interactome-based principles in the ICU.

### 3.6 Multi-axial crosstalk: synergistic and antagonistic intertwining within the interactome

A defining characteristic of the gut interactome is that its functional outcomes are thought to emerge from continuous, multi-axial cross-talk among the SCFA, AhR, PRR, and bile acid pathways, which severe trauma reconfigures from a homeostatic web into a destructive cascade of synchronized failure. Under homeostasis, these axes are believed to operate via tight molecular synergy; microbial butyrate, acting as HDACs inhibitor (Schulthess et al., 2019), has been shown to upregulate the transcriptional responsiveness of host cells to AhR ligands (Qiu et al., 2012), and may thereby amplify IL-22 and Reg3γ expression (Qiu et al., 2012; Zelante et al., 2013). Simultaneously, physiologic hypoxia sustained by anaerobic metabolism stabilizes HIF-1α to reinforce tight junctions (Peng et al., 2009; Yu et al., 2018; Wang et al., 2024). A critical tonic antagonism is thought to be maintained where SCFA and AhR signaling continuously suppress macrophage NF-κB translocation, counteracting basolateral TLR4 hyperactivation (Abreu, 2010; Quintana and Sherr, 2013; Schulthess et al., 2019), while secondary bile acids activate TGR5 to inhibit NLRP3 inflammasome assembly (Yan et al., 2025). Following severe trauma, this integrated network is proposed to invert into a lethal, self-perpetuating loop. Acute SCFA starvation has been associated with an epithelial ATP crisis that is thought to disrupt tight junctions (Peng et al., 2009; Donohoe et al., 2011), thereby permitting an influx of LPS and necrotic DAMPs (Jiang et al., 1995; Lord et al., 2014). Upon encountering exposed basolateral TLR4, these interleaved danger signals are thought to ignite a massive pro-inflammatory cascade via the MyD88/NF-κB pathway (Poltorak et al., 1998). This hyper-inflammation is suggested to repress host Tph1 (Yano et al., 2015), thereby potentially starving the AhR axis and silencing IL-22-driven antimicrobial peptide secretion (Sonnenberg et al., 2011; Sun et al., 2023). In the absence of competitive suppression, the blooming pathobiome is associated with eradication of BSH-active and 7α-dehydrogenase-encoding anaerobes, halting secondary bile acid conversion (Ridlon et al., 2006; Kullberg et al., 2025). The resulting accumulation of primary conjugated bile acids is reported to exert a detergent effect that may dissolve epithelial membranes and amplifies apoptosis (Islam et al., 2011), leaking more danger signals to refuel basolateral TLR4 activation and potentially locking the post-traumatic gut interactome into a self-perpetuating cycle that is thought to drive systemic MODS (Klingensmith and Coopersmith, 2016; Ma et al., 2021).

## 4 From molecular targets to integrated therapeutic strategies

In response to the ecological storm unleashed within the gut by severe trauma, the therapeutic goal must shift from merely protecting the mucosa toward the multi-dimensional repair and remodeling of the gut interactome.

### 4.1 Preclinical mechanistic synthesis of metabolic and target-directed pathways

At the cellular level, SCFAs, predominantly butyrate, are conceptualized as foundational metabolic fuels that interact with colonocyte GPR41/GPR43 receptors to regulate mucosal inflammatory homeostasis *in vitro* (Schulthess et al., 2019). Preclinical evaluation suggests that mitochondrial β-oxidation of these substrates supports baseline enterocyte ATP requirements, thereby maintaining the functional alignment of tight junction barriers (Donohoe et al., 2011). Concurrently, the activation of the intracellular AhR via microbiota-derived indole metabolites represents a key signaling node; this ligand-dependent translocation is thought to promote IL-22 transcription in ILC3s, which is implicated in the downstream expression of protective antimicrobial peptides (Reg3γ and Reg3β) (Qiu et al., 2012; Zelante et al., 2013). To counteract pathobiome expansion without provoking antimicrobial resistance, non-lethal virulence control strategies have been evaluated. Specifically, the deployment of high-molecular-weight polymers, such as phosphate-containing polyethylene glycol (Pi-PEG), is suggested to represent a proof-of-concept physical shielding mechanism that may disrupt the ability of opportunistic pathogens to sense host stress parameters, and is thought to suppress phenotypic virulence switches in controlled rodent models (Hyoju et al., 2022).

### 4.2 Preclinical neuro-immune and compound-mediated target networks

Horizontal integration across the gut interactome involves parallel neuro-immune and humoral pathways characterized in basic research models. Preclinical evidence indicates that sennoside A, a primary anthraquinone glycoside derived from Dachengqi Decoction (DCQD), possesses the structural capacity to target the LasR transcriptional regulator of *Pseudomonas aeruginosa*, executing quorum quenching to attenuate virulence factor expression at sub-inhibitory concentrations *in vitro* (Han et al., 2022). In parallel, autonomic modulation of mucosal immunity is frequently modeled via peripheral bioelectronic pathways. Controlled rodent assays suggest that electrical stimulation at the Zusanli (ST36) acupoint targets the host vagal-adrenal axis, which is thought to prompt localized acetylcholine secretion (Song et al., 2021; Yang et al., 2021). This neurotransmitter is postulated to engage macrophage α7 nicotinic acetylcholine receptors (α7nAChR), which may subsequently suppress downstream NF-κB nuclear translocation and modulate macrophage inflammatory polarization phenotypes (Yang et al., 2021; Mengzhu et al., 2022). Collectively, these multi-target interventions synthesize a complex network of preclinical targets capable of modifying individual dialogue nodes within the disrupted interactome framework.

### 4.3 Stage-specific clinical recommendations for ecological restoration

Translating metabolic and ecological interventions into the intensive care unit requires strict categorization according to the clinical hierarchy of evidence and stage-specific critical care pathophysiology. To ensure transparency in evidence grading throughout this section, we adopted a modified hierarchy adapted from the Oxford Centre for Evidence-Based Medicine (OCEBM) criteria (Howick et al., 2011), tailored to the critical care and trauma setting. Level I denotes consistent findings from large-scale randomized controlled trials (RCTs), systematic reviews, or meta-analyses in human populations. Level II indicates evidence from prospective cohort studies, controlled non-randomized trials, or small-scale RCTs. Level III encompasses retrospective cohort studies, case–control studies, or large case series. Level IV refers to preclinical studies (animal models or *in vitro* experiments), small case series, or expert consensus based on pathophysiological reasoning. Level V denotes extrapolated evidence from non-trauma populations when no direct trauma-specific human data are available.

#### 4.3.1 Acute phase adjustments (FMT vs. fiber)

During the hyper-acute phase of severe trauma, Fecal Microbiota Transplantation (FMT) is generally considered contraindicated due to the theoretical risk of passive bacterial translocation across a severely ischemic, hyper-permeable mucosal barrier. This concern is supported by indirect evidence from animal models of hemorrhagic shock demonstrating increased bacterial translocation under conditions of gut hypoperfusion (Klingensmith and Coopersmith, 2016), as well as clinical consensus in critically ill populations (Hyoju et al., 2022). However, direct human data on FMT specifically in acute trauma patients are currently lacking, and the contraindication is based primarily on pathophysiological reasoning and expert opinion rather than trauma-specific clinical trials. Evidence Grade: Level IV (preclinical models + expert consensus; direct human trauma data unavailable).

#### 4.3.2 Subacute phase niche reset

In the subacute window characterized by stable hemodynamics but severe, broad-spectrum antibiotic-induced dysbiosis, FMT has been proposed as a potential strategy to restore microbial community structure via competitive niche exclusion. This recommendation is primarily supported by a rodent model of traumatic brain injury demonstrating that FMT ameliorates neurological deficits and restores gut microbial diversity (Du et al., 2021), as well as human data from patients with recurrent *Clostridium difficile* infection showing that donor fecal infusion effectively re-establishes a healthy microbiota (van Nood et al., 2013). The conceptual framework for FMT in the trauma setting has also been discussed in recent critical care reviews (Cao et al., 2025). However, direct clinical evidence for FMT specifically in trauma patients with antibiotic-induced dysbiosis is currently lacking, and the recommendation remains largely extrapolated from non-trauma populations and preclinical models. Evidence Grade: Level IV (preclinical models + human data from non-trauma populations; direct trauma-specific evidence currently unavailable).

#### 4.3.3 Recovery phase niche construction

During the stable recovery and rehabilitation phase, the systematic administration of dietary fiber and targeted prebiotics has been proposed to support endogenous niche construction, potentially fueling the metabolic rekindling of native SCFA-producing obligate anaerobes to support long-term mucosal barrier repair. This recommendation is supported by human studies in critically ill and surgical populations demonstrating that enteral nutrition with fiber-containing formulas is associated with improved gut barrier function and reduced infectious complications (Taylor et al., 2016; Reintam Blaser et al., 2017), as well as a mouse model of microbiome recovery showing that diet outperforms microbial transplant in restoring microbial community structure (Kennedy et al., 2025) and human studies in non-trauma surgical populations suggesting that preoperative dietary prehabilitation may improve outcomes (Keskey et al., 2022). However, direct evidence specifically in trauma patients remains limited, and extrapolation from these populations should be interpreted with caution. Evidence Grade: Level V (extrapolated from non-trauma ICU/surgical populations + preclinical models; direct trauma-specific human data currently lacking).

### 4.4 Evidence-graded clinical translation of integrative medicine countermeasures

The clinical deployment of traditional pharmacological formulas and neuromodulatory techniques requires validation through rigorous clinical trial evidence rather than reliance on preclinical findings alone, before translation to the bedside. It should be noted, however, that the evidence base for integrative medicine interventions in trauma populations remains preliminary, and the following recommendations should be interpreted with appropriate caution.

#### 4.4.1 Clinical efficacy of modified DCQD

The integration of modified DCQD for managing post-traumatic gastrointestinal dysfunction and intra-abdominal hypertension (IAH) is supported by preliminary clinical evidence. Prospective cohort data and single-center controlled critical care pilots suggest that early enteral administration of modified DCQD within 24–48 h of ICU admission has been associated with a significant acceleration of intestinal peristalsis recovery and decreased feeding intolerance (Li et al., 2020; Tian et al., 2020). Furthermore, sequential blood profiling suggests that these clinical benefits are accompanied by a time-dependent reduction in circulating plasma diamine oxidase (DAO) and I-FABP levels, which is indicative of enterocyte salvage in human cohorts (Piton and Capellier, 2016).

Several important limitations should be acknowledged when interpreting these findings. First, the majority of clinical studies on DCQD in the trauma setting have been conducted by a single research group, and independent replication from other centers is currently lacking. Second, sample sizes in the reported cohorts remain relatively modest, limiting the precision of effect estimates. Third, the observed clinical benefits are based largely on surrogate biomarkers (DAO and I-FABP) rather than patient-centered outcomes such as mortality, infectious complications, or length of ICU stay. While the existing data are encouraging, they should be interpreted as preliminary and hypothesis-generating rather than conclusive. Larger, multi-center randomized controlled trials with predefined hard clinical endpoints are required before definitive recommendations can be made. Evidence Grade: Level III (prospective cohort studies, single-center; independent multi-center replication currently lacking; endpoints based on surrogate biomarkers rather than patient-centered outcomes).

#### 4.4.2 Clinical efficacy of electroacupuncture

The use of electroacupuncture at specific acupoints (such as ST36 and Neiguan, PC6) to resolve critical illness-associated paralytic ileus is supported by clinical evidence from randomized controlled trials in post-surgical and mixed ICU populations. Standardized RCTs and systematic reviews indicate that active EA is associated with significantly shortened duration of post-operative ileus and reduced mean duration of mechanical ventilation in intensive care settings (Wang et al., 2015; Yang et al., 2021). This pro-motility effect correlates with systemic anti-inflammatory protection, as human trial datasets suggest a robust, time-dependent suppression of circulating pro-inflammatory cytokines, including TNF-α and IL-6, following standardized electronic stimulation (Yang et al., 2021).

Nevertheless, several caveats should be acknowledged when interpreting these findings. Most existing trials have been conducted in post-operative rather than trauma-specific populations, and whether the observed benefits generalize to polytrauma patients with hemorrhagic shock or severe tissue injury remains to be established. Additionally, sample sizes across the available studies remain moderate, and the heterogeneity of EA protocols (stimulation parameters, treatment duration, and concomitant therapies) complicates direct comparison and standardization. While the current evidence is promising, trauma-specific, multi-center RCTs with patient-centered endpoints are needed to confirm the efficacy and optimal application of EA in this population. Evidence Grade: Level II (multi-center RCTs in post-surgical and mixed ICU populations; trauma-specific data currently limited).

### 4.5 An integrated framework of therapeutic strategies

**FIGURE 3:**
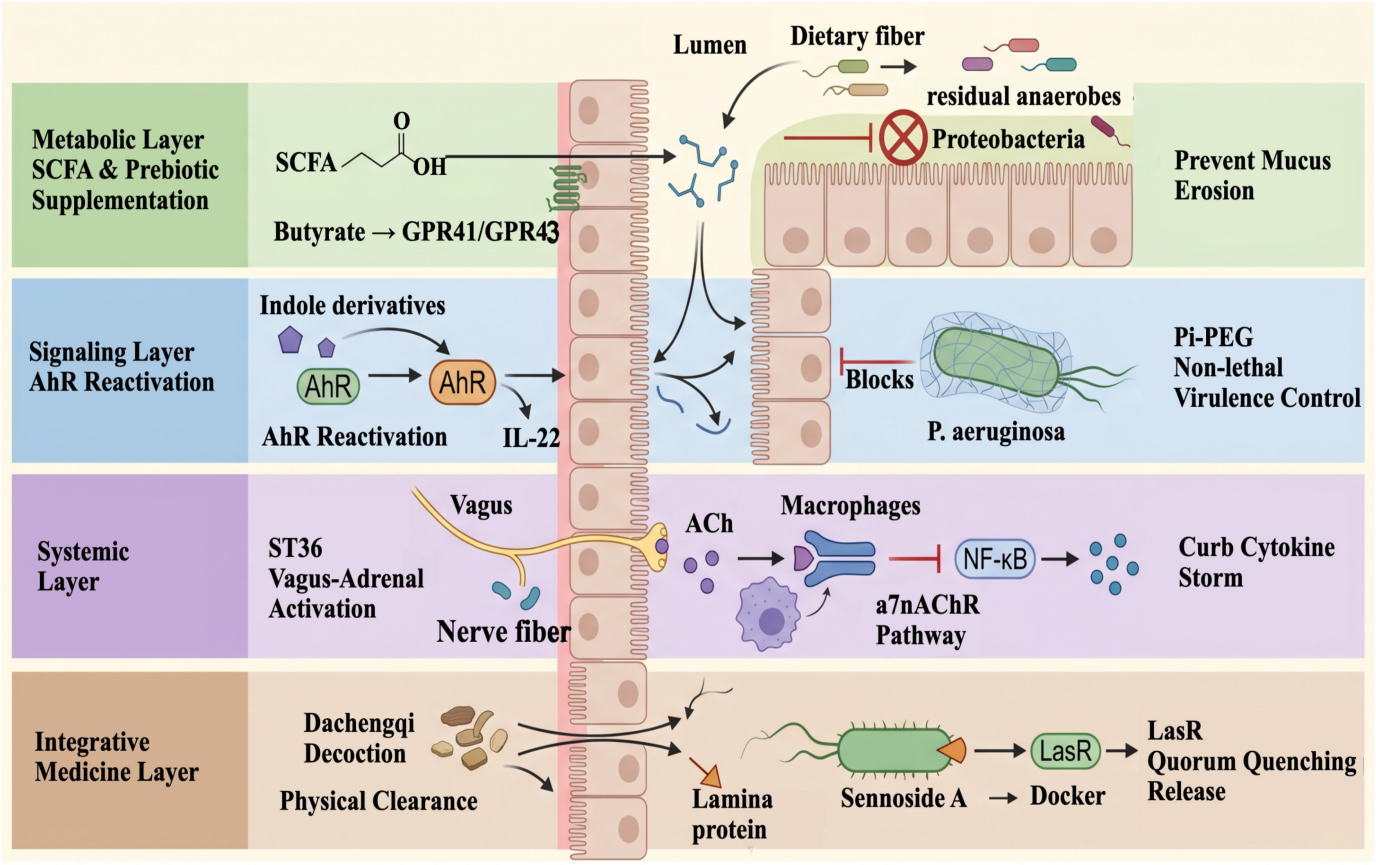
Integrated framework for remodeling the post-traumatic gut interactome. A flat multi-layered diagram mapping the structured molecular interventions designed to reverse barrier failure and dysbiosis, directly corresponding to the therapeutic levels detailed in Table 2. Metabolic Layer is intended to restore nutritional homeostasis via exogenous butyrate targeting epithelial GPR41/GPR43 to re-establish downregulatory mechanisms, while dietary fiber fuels beneficial anaerobes to prevent starvation-induced mucus erosion by Proteobacteria. Signaling Layer is designed to reactivate mucosal defenses and non-lethal pathogen control via indole-mediated AhR nuclear translocation for IL-22 expression, concurrently deploying Pi-PEG polymer mesh to encapsulate *Pseudomonas aeruginosa* and block host stress signals from activating its virulence switch. Systemic Layer is thought to regulate the neural-immune axis via ST36 electroacupuncture activation of the vagal-adrenal axis to release acetylcholine (ACh), which binds to macrophage α7nAChR to suppress the downstream NF-κB cascade and curb the cytokine storm. Integrative Medicine Layer is designed to execute multi-target clearance and quorum quenching via Dachengqi Decoction for physical evacuation of lumens, while its core component, Sennoside A, targets the LasR transcriptional regulator of *Pseudomonas aeruginosa* to block virulence factor release at sub-inhibitory concentrations. Created with BioGDP.com (Jiang et al., 2025).

As shown in Figure 3 and Table 2, comprehensive interventions are designed to reconstruct the intestinal dialogue from various dimensions.

**TABLE 2:** Evidence-graded framework for remodeling the post-traumatic gut interactome.

Intervention layer	Preclinical molecular target	Clinical strategy and ICU translation workflow	Evidence grade	Key references
Metabolic layer	Butyrate supplies colonic ATP via β-oxidation; binds colonocyte GPR41/GPR43	Enteral SCFA formulas to fuel enterocytes; stage-specific dietary fiber during stable recovery phases	Level V	Schulthess et al., 2019; Kennedy et al., 2025
Systemic layer	Acetylcholine binds macrophage α7nAChR to repress the NF-κB cascade	Standardized electroacupuncture (ST36/PC6) to mitigate paralytic ileus and mechanical ventilation duration	Level II	Song et al., 2021; Yang et al., 2021
Integrative layer	Purging of pathobiome load; sub-inhibitory LasR quorum quenching	Enteral modified DCQD within 24–48 h to mitigate intra-abdominal hypertension and decrease plasma DAO/I-FABP	Level III	Piton and Capellier, 2016; Li et al., 2020; Tian et al., 2020; Han et al., 2022
Ecological layer	Wholesale introduction of healthy donor community; competitive pathobiont exclusion	FMT deployment restricted exclusively to subacute antibiotic-induced dysbiosis; strictly contraindicated in acute shock	Level IV	van Nood et al., 2013; Du et al., 2021; Cao et al., 2025
Signaling layer	Indole-mediated AhR activation drives IL-22; Pi-PEG encapsulates pathogens	Targeted receptor reactivation and non-lethal virulence shielding to prevent phenotypic switches *in vitro*	Level IV	Qiu et al., 2012; Zelante et al., 2013; Mengzhu et al., 2022

## 5 New clinical pathways and future directions

### 5.1 Dynamic functional omics: constructing the decision-making cornerstone of a real-time clinical pathway

**FIGURE 4:**
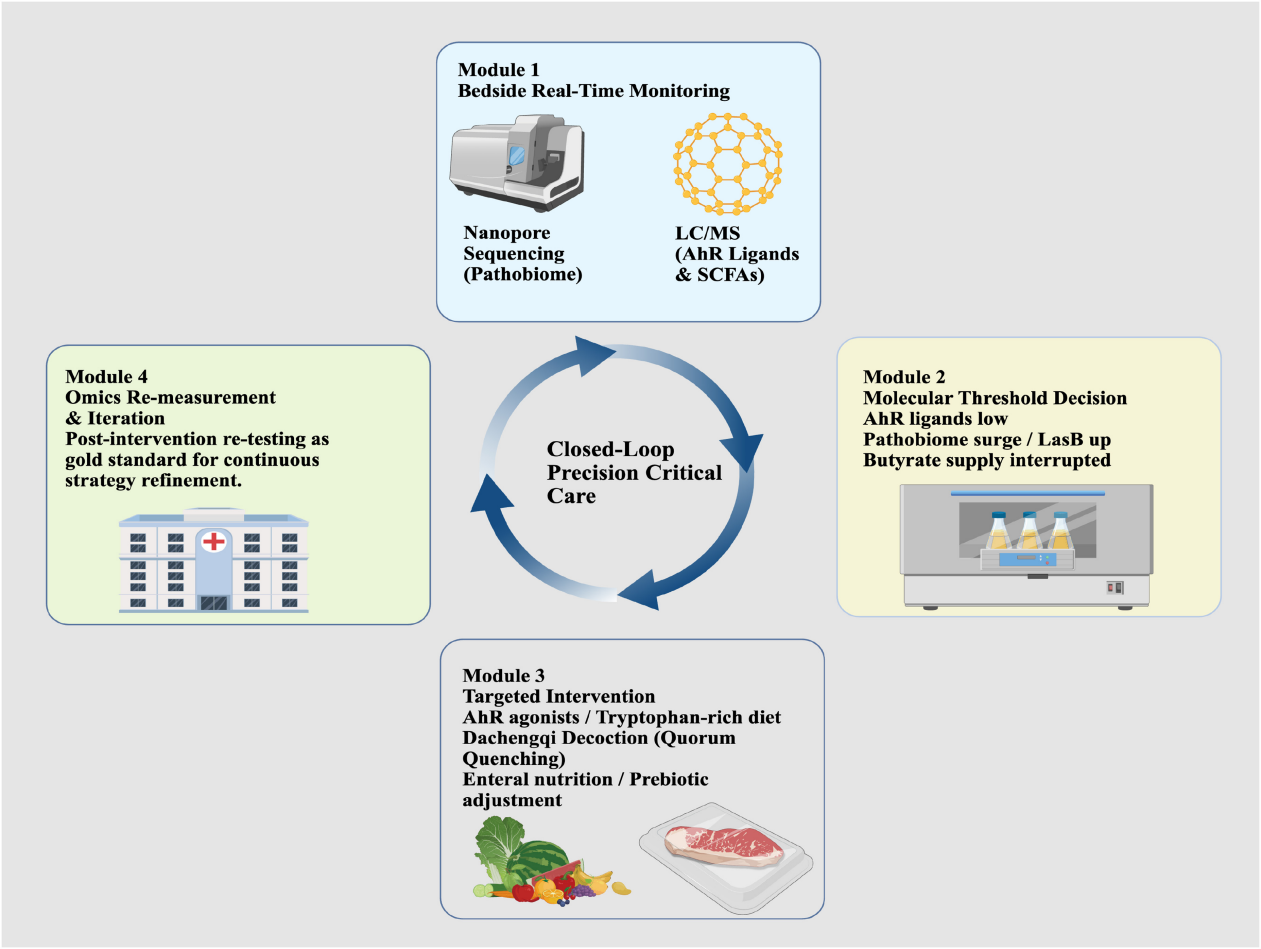
Real-time clinical pathway based on dynamic functional omics monitoring. A flat closed-loop diagram visualizing the transition to proactive precision critical care via sequential sensing, threshold assessment, and targeted intervention. Module 1: Bedside Real-Time Monitoring – Establishes the continuous sensing input layer via bedside Nanopore sequencing for rapid pathobiome decoding and LC/MS monitoring for tracking AhR ligands and SCFA curves. Module 2: Molecular Threshold Decision – Operates as the central algorithmic matrix, evaluating multi-omics data against predetermined thresholds to triage critical AhR ligand depletion, pathobiome surges with LasB upregulation, or butyrate supply interruptions. Module 3: Targeted Intervention – Executes precision clinical pathways tailored to threshold alarms, routing patients into immediate AhR agonist rescue, Dachengqi Decoction-mediated quorum quenching, or prebiotic formula adjustments. Module 4: Omics Re-measurement and Iteration – Drives the post-intervention feedback loop via follow-up multi-omics re-testing to continuously evaluate therapeutic efficacy and refine iterative treatment strategies back into Module 1. Created with BioGDP.com (Jiang et al., 2025).

Future trauma care may benefit from moving beyond static clinical assessments and could shift toward a real-time clinical pathway driven by dynamic omics data. A core feature of this proposed pathway is leveraging high-throughput monitoring technologies to convert otherwise imperceptible perturbations within the gut interactome into quantifiable triggers for intervention. This approach could facilitate a paradigm shift from reactive treatment toward proactive regulation, as structurally mapped out in the multi-layered framework of Figure 4.

#### 5.1.1 Rapid sensing: the golden window from sample to decision

The realization of a real-time clinical pathway would be predicated on the iteration of monitoring technologies. Nanopore long-read sequencing has been reported to allow clinicians to perform a rapid analysis of 16S rRNA or the metagenome from fecal samples within hours, enabling the real-time identification of surges in pathobiome abundance (You et al., 2025). Concurrent bedside metabolic monitoring, integrating rapid liquid chromatography or mass spectrometry, could permit the real-time tracking of AhR ligands, such as indole-3-aldehyde and indole-3-acetic acid, and SCFAs in both fecal and blood samples. This multi-dimensional, immediate feedback forms the sensory foundation of the pathway.

#### 5.1.2 Decision matrix: dynamic intervention based on molecular thresholds

The real-time clinical pathway could guide therapeutic decisions via a decision matrix model grounded in specific molecular monitoring thresholds. The AhR alert and reactivation pathway could be triggered when monitored concentrations of AhR ligands in the gut are found to fall below a predetermined threshold, a condition that signals the impending failure of mucosal immune surveillance. This would potentially activate a targeted rescue protocol using AhR agonists or a tryptophan-rich dietary intervention to rebuild the immune barrier before bacterial translocation becomes established (Qiu et al., 2012; Zelante et al., 2013). The pathobiome expansion and Tongfu pathway could be triggered by two conditions (Munley et al., 2023a). The first is an exponential increase in the abundance of the pathobiome, particularly *Pseudomonas aeruginosa* or Enterobacteriaceae. The second is the upregulation of specific virulence genes, such as LasB (Williams et al., 2000). The pathway directs the administration of a multi-target herbal formula, such as the Dachengqi decoction. This intervention is intended to achieve both physical clearance and quorum quenching, thereby weakening the pathogenic advantage without generating the selective pressure associated with antibiotics (Han et al., 2022). The metabolic reserve and recycling pathway would continuously assess SCFA levels. Upon detecting an interruption in butyrate supply, the pathway would immediately trigger an adjustment of the enteral nutrition formula or supplementation of prebiotics. This step is thought to prevent pathogenic bacteria from eroding the mucus layer under conditions of ecological starvation (Schulthess et al., 2019; Kennedy et al., 2025).

#### 5.1.3 Clinical translational value: toward precision critical care

The establishment of this pathway could integrate previously fragmented interventions, including herbal medicine, nutritional support, and neuromodulation, into a single evidence-based, dynamic framework. This integration might enable personalized intervention that avoids the pitfalls of a one-size-fits-all approach, such as the blanket use of prophylactic antibiotics, and potentially reduce iatrogenic secondary injury. Furthermore, it could permit prospective treatment by using molecular alerts to initiate pre-emptive therapy before clinical symptoms, such as fever or ileus, become manifest. Finally, repeated omics measurements following intervention could serve as a gold standard for evaluating the functional recovery of the interactome, facilitating the continuous iteration of the treatment strategy.

#### 5.1.4 Translational hurdles: engineering bottlenecks and future optimization

Transitioning this closed-loop dynamic functional omics pathway into routine critical care is likely to face four primary logistical and biophysical bottlenecks. First, sequencing and mass spectrometry costs currently remain prohibitive (Johnson et al., 2016). Second, volatile bedside fecal and blood transcripts and metabolites are subject to rapid enzymatic degradation (Siskos et al., 2017). Third, the several-hour detection cycle may not align with the hyper-acute golden hours of resuscitation (Klingensmith and Coopersmith, 2016). Fourth, intense inter-individual baseline heterogeneity complicates the establishment of unified judgment thresholds (Gilbert et al., 2018). To overcome these hurdles, future optimization may need to integrate microfluidic point-of-care testing (POCT) chips and automated bedside stabilization buffers to drastically reduce assay costs, preserve fragile molecular cargo, and compress turnaround times to under an hour (Seymour et al., 2017). Concurrently, deploying artificial intelligence and machine learning algorithms onto large-scale, multi-center longitudinal datasets has been proposed as a potential path to modeling adaptive, patient-specific threshold matrices. This approach transforms the current static theoretical framework into a precision-guided critical care engine (Komorowski et al., 2018).

### 5.2 Transition from pathogen clearance to symbiotic equilibrium

A central implication of interactome theory is that future therapeutic strategies may need to shift from pure pathogen clearance to restoration of functional balance between host and microbiota. The limitations of direct sterilization via antibiotics as the sole intervention have become increasingly apparent. A multicenter cohort study provides important epidemiological evidence, indicating that patients with a history of antibiotic exposure during a previous hospitalization had a 65% increased risk for sepsis within 90 days of discharge (Baggs et al., 2018). A key element of this restorative strategy is thought to be inhibition of the transformation of virulence phenotypes in the pathogenic group rather than complete eradication of the microbial community. Alverdy’s group has tested the concept in animal models. Oral administration of Pi-PEG, a phosphate-rich polymer, successfully prevented surgical site infections. This protection was associated with inhibition of virulence expression during bacterial migration from the gut to the wound, without altering the structure of the gut microbiota (Hyoju et al., 2022). It is noteworthy that the active ingredients extracted from traditional Chinese medicine appear to share a common pharmacological logic with this modern strategy. For example, while sennoside A has been reported to inhibit the quorum-sensing system of *Pseudomonas aeruginosa* by targeting its LasR transcriptional regulatory proteins to disarm virulence at subinhibitory concentrations *in vitro* (Han et al., 2022), this interaction is currently best interpreted as a proof-of-concept paradigm, given the substantial pharmacokinetic and bio-distribution hurdles encountered *in vivo*. This series of integrated treatment strategies based on specific molecular targets suggests a possible future direction for the realization of interactome oriented precision intervention.

## 6 Discussion

### 6.1 Synthesis of core framework and contributions

The core model advanced in this review is the transition from a passive, linear model of intestinal barrier failure to a dynamic, multi-compartmental collapse of the gut interactome following severe trauma (Klingensmith and Coopersmith, 2016; Cao et al., 2025). Rather than viewing the gut merely as an anatomical victim of hemorrhagic shock or a passive reservoir of translocating pathogens, this framework proposes to conceptualize post-traumatic critical illness as a profound reprogramming of bidirectional, reciprocal chemical dialogues. These dialogues occur among host immune sentinels, commensal metabolic networks, and opportunistic pathogens. This work systematically reviews the post-traumatic collapse across four synchronized molecular axes: the energetic failure of SCFAs, the starvation of AhR signaling, the cross-activation of PRRs by interleaved DAMPs and PAMPs, and the cytotoxic shift of the bile acid pool. Through this mechanistic lens, the review attempts to bridge microscopic molecular alterations with macroscopic remote organ failure. Furthermore, by framing iatrogenic critical care interventions as an intensive selective pressure that must be balanced against their life-saving necessity (Dethlefsen et al., 2008; Baggs et al., 2018), this review draws attention to a fundamental therapeutic paradox: while these interventions are indispensable in acute trauma care, they may inadvertently accelerate virulence expression within the emergent pathobiome. This paradox underscores the need for judicious application and active mitigation strategies, such as antibiotic stewardship, multimodal analgesia, and early enteral nutrition, rather than wholesale avoidance of these critical interventions.

### 6.2 Comparative innovations and supplementary value

When contrasted with previously published reviews in the fields of surgical microbiomics and critical care immunology, several distinct points of emphasis emerge. Prior literature has predominantly operated within a descriptive model, with a strong focus on static taxonomic shifts such as alpha-diversity reduction or altered Firmicutes-to-Bacteroidetes ratios, or characterizing physical barrier breakdown in isolation (Munley et al., 2023b; Sun et al., 2024). While valuable, these frameworks do not always fully account for key clinical paradoxes, such as distant organ infections occurring in the strict absence of bacteremia (Merkow et al., 2015), or the rapid onset of acute lung injury before systemic infectious markers manifest. This review seeks to supplement existing literature by introducing a tri-directional interactome model that treats pathogen virulence not as an intrinsic genetic trait, but as an emergent phenotype dynamically triggered by host stress cues and microenvironmental sieves. Crucially, while previous works remain largely descriptive, this review attempts to move beyond mechanistic cataloging toward proposing a multi-layered, target-specific therapeutic framework. By integrating Western bioengineering strategies (Hyoju et al., 2022) with high-quality evidence from traditional Chinese medicine compounds (Han et al., 2022) and neuro-immune neuromodulation (Yang et al., 2021). This work proposes a clinical translation framework that may contribute to narrowing the gap between benchside systems biology and clinical precision medicine (Babrowski et al., 2013).

### 6.3 General limitations of the current field

Despite the rapid evolution of interactome theory, several systemic limitations persist across current research methodologies that currently hinder immediate clinical translation. First, the vast majority of mechanistic insights are derived from highly controlled rodent models which may not fully replicate human complexity, including the non-hepatic bypass of the gut-lymph axis and M1 pro-inflammatory polarization of alveolar macrophages (Kojima et al., 2018; Ma et al., 2021). Laboratory rodents do not fully replicate the physiological complexities of human trauma patients, who often present with extreme heterogeneity in age, pre-existing comorbidities, diverse tissue injury patterns, and vastly different fluid resuscitation volumes. Second, current sampling methodologies remain predominantly cross-sectional or rely on low-resolution timelines. Because post-traumatic interactome reprogramming occurs with hyper-acute kinetics within the golden hours of initial resuscitation, standard retrospective fecal sampling may fail to capture the volatile, real-time metabolic and transcriptomic shifts occurring at the mucosal-microbiome interface (de Haan et al., 2009). Third, there remains a significant gap in functional validation. Although high-throughput 16S rRNA sequencing provides taxonomic profiles, current studies rely heavily on predicted functional pathways rather than on direct, high-resolution metatranscriptomic and untargeted metabolomic validation of active dialogue nodes *in vivo* (Knight et al., 2018).

### 6.4 Controversies and unresolved issues in trauma research

The application of interactome and pathobiome theories to trauma medicine has sparked several critical controversies that warrant explicit acknowledgment and unresolved debates. A primary source of contention lies in the definition of a universal baseline healthy or resilient interactome (Eckburg et al., 2005). Given the immense inter-individual variations in baseline microbiome architecture driven by diet, geography, and genetics, predicting which patients possess an interactome that may resist post-traumatic virulence switches remains highly elusive. Another profound clinical controversy centers around the Eradication vs. Restorative paradox. Although the detrimental effects of anti-anaerobic broad-spectrum antibiotics on colonization resistance are widely recognized (Chanderraj et al., 2023; Kullberg et al., 2025), establishing a precise safety threshold for withholding empiric antimicrobial therapy in suspected post-traumatic sepsis remains an intensely debated dilemma in ICU guidelines (Baggs et al., 2018). Finally, the role of the gut-lymph axis as a primary vehicle for distant organ failure in human clinical scenarios remains highly controversial, as discussed in section “3.3 Dual-channel conduction of remote organ injury: the gut-lymph axis” and illustrated in Figure 2. While elegant rodent models have shown that blocking mesenteric lymph can prevent lung injury under controlled conditions (Deitch, 2010), this pathway remains a hypothesis derived primarily from animal studies. Obtaining direct, causal validation in human trauma cohorts presents extreme ethical and logistical barriers. Sampling mesenteric lymph or isolating intact, gut-derived toxic exosomes from circulating human blood under acute resuscitation conditions is exceptionally difficult, leaving the exact contribution of this non-hepatic bypass in human patients an open question that requires further investigation. Therefore, while the gut-lymph axis offers a compelling mechanistic framework to guide experimental design and biomarker discovery, its clinical significance should be interpreted with appropriate caution until validated in human studies.

## 7 Conclusion

The disruption of the gut interactome after severe trauma may be viewed as a complex molecular process that is thought to involve the collaborative evolution of host energy metabolism crisis, immune signal imbalance, microbial metabolic failure, and pathogen virulence transformation. Decoding this molecular communication network may provide potential targets for precise interventions. These include SCFA supplementation for potential restoration of epithelial energy supply and immunomodulatory function, aryl hydrocarbon receptor ligand reactivation to support mucosal barrier integrity, multi-targeted traditional Chinese medicine compounds for potential clearance of pathogenic groups and modulation of their virulence expression (though current evidence remains preliminary and requires multi-center validation), and acupuncture neuromodulation for modulation of excessive inflammatory responses. The core direction of interactome-based precision intervention is proposed to shift from merely eliminating pathogens toward restoring the functional balance between host and microbiota. This approach is intended to support symbiotic homeostasis through modulation of virulence transformation in pathogenic groups, while recognizing that iatrogenic interventions must be carefully balanced against their life-saving necessity. A deeper molecular understanding of interactome evolution during the whole process of disease occurrence and treatment response may lay the foundation for more personalized and targeted integrated treatment in critical care settings.

## Glossary of key terms

Interactome: The integrated, multi-directional signaling network among the host immune system, gut microbiota, and local microenvironment; its collapse under severe trauma drives the transition from symbiotic homeostasis to systemic inflammation and remote organ dysfunction.
Pathobiome: A disease-associated microbial community selected from the endogenous commensal pool by environmental pressures (e.g., oxidative stress, antibiotics), characterized by facultative anaerobe overgrowth and virulence upregulation. It represents a pathological end-state of interactome disruption.
Tri-directional dialogue: The reciprocal biochemical communication among host immunity, microbial metabolism, and microenvironmental stressors that determines whether the interactome maintains homeostasis or collapses into a pathological state.
AhR (Aryl hydrocarbon receptor): A transcription factor activated by microbiota-derived tryptophan metabolites; drives IL-22 production and maintains mucosal barrier integrity.
DAMPs (Damage-associated molecular patterns): Host-derived molecules (e.g., HMGB1, mtDNA, ATP) released from injured cells that activate innate immunity and trigger sterile inflammation.
DAO (Diamine oxidase): An epithelial enzyme whose elevated circulating levels serve as a biomarker of intestinal mucosal injury.
DCQD (Dachengqi Decoction): A traditional Chinese medicine formula used to promote gastrointestinal motility; its component sennoside A exerts quorum-quenching effects against *Pseudomonas aeruginosa*.
Dysbiosis: A descriptive term for any compositional shift of the gut microbiota away from a healthy baseline, which may or may not progress to pathobiome formation.
FMT (Fecal microbiota transplantation): Transfer of healthy donor stool to restore microbial community structure; contraindicated in acute trauma due to translocation risk.
FXR/FGF19: A bile-acid-activated nuclear receptor that induces FGF19 secretion to suppress hepatic bile acid synthesis, maintaining bile acid pool homeostasis.
I-FABP (Intestinal fatty acid-binding protein): A cytosolic protein released from injured enterocytes; serves as an early biomarker of intestinal epithelial damage.
MODS (Multiple organ dysfunction syndrome): Progressive failure of two or more organ systems in critically ill patients, often driven by gut-derived inflammatory mediators.
PAMPs (Pathogen-associated molecular patterns): Conserved microbial motifs (e.g., LPS, flagellin) recognized by host PRRs to initiate immune responses.
PRRs (Pattern recognition receptors): Host sensors (TLRs and NLRs) that detect PAMPs and DAMPs; their spatial segregation across the epithelial barrier maintains immune tolerance while enabling pathogen vigilance.
SCFAs (Short-chain fatty acids): Microbial metabolites (acetate, propionate, butyrate) produced by fiber fermentation; serve as epithelial energy sources, HDAC inhibitors, and immunomodulatory signaling molecules.
TGR5: A secondary bile acid receptor on immune and enteroendocrine cells; its activation inhibits NLRP3 inflammasome and promotes GLP-1 secretion.
